# Antifungal Activity of Isolated Compounds from the Leaves of *Combretum erythrophyllum* (Burch.) Sond. and *Withania somnifera* (L.) Dunal against *Fusarium* Pathogens

**DOI:** 10.3390/molecules26164732

**Published:** 2021-08-05

**Authors:** Hlabana Alfred Seepe, Tselane Geneva Ramakadi, Charity Mekgwa Lebepe, Stephen O. Amoo, Winston Nxumalo

**Affiliations:** 1 Agricultural Research Council—Vegetables, Industrial and Medicinal Plants, Roodeplaat, Private Bag X293, Pretoria 0001, South Africa; amoos@arc.agric.za; 2 Department of Chemistry, University of Limpopo, Private Bag X1106, Sovenga 0727, South Africa; tselane.ramakadi@ul.ac.za (T.G.R.); charity.lebepe@gmail.com (C.M.L.); 3 Indigenous Knowledge Systems Centre, Faculty of Natural and Agricultural Sciences, North-West University, Private Bag X2046, Mmabatho 2735, South Africa; 4 Department of Botany and Plant Biotechnology, Faculty of Science, University of Johannesburg, P.O. Box 524, Auckland Park 2006, South Africa

**Keywords:** apigenin, maslinic acid, natural products, phytotoxicity, purification, salvigenin, withaferin A

## Abstract

Crop diseases caused by *Fusarium* pathogens, among other microorganisms, threaten crop production in both commercial and smallholder farming. There are increasing concerns about the use of conventional synthetic fungicides due to fungal resistance and the associated negative effects of these chemicals on human health, livestock and the environment. This leads to the search for alternative fungicides from nature, especially from plants. The objectives of this study were to characterize isolated compounds from *Combretum erythrophyllum* (Burch.) Sond. and *Withania somnifera* (L.) Dunal leaf extracts, evaluate their antifungal activity against *Fusarium* pathogens, their phytotoxicity on maize seed germination and their cytotoxicity effect on Raw 264.7 macrophage cells. The investigation led to the isolation of antifungal compounds characterized as 5-hydroxy-7,4′-dimethoxyflavone, maslinic acid (21-hydroxy-3-oxo-olean-12-en-28-oic acid) and withaferin A (4β,27-dihydroxy-1-oxo-5β,6β-epoxywitha-2-24-dienolide). The structural elucidation of the isolated compounds was established using nuclear magnetic resonance (NMR) spectroscopy, mass spectroscopy (MS) and, in comparison, with the available published data. These compounds showed good antifungal activity with minimum inhibitory concentrations (MIC) less than 1.0 mg/mL against one or more of the tested *Fusarium* pathogens (*F. oxysporum, F. verticilloides, F. subglutinans, F. proliferatum, F. solani, F. graminearum, F. chlamydosporum* and *F. semitectum*). The findings from this study indicate that medicinal plants are a good source of natural antifungals. Furthermore, the isolated antifungal compounds did not show any phytotoxic effects on maize seed germination. The toxicity of the compounds **A** (5-hydroxy-7,4′-dimethoxyflavone) and **AI** (4β,27-dihydroxy-1-oxo-5β,6β-epoxywitha-2-24-dienolide) was dose-dependent, while compound **B** (21-hydroxy-3-oxo-olean-12-en-28-oic acid) showed no toxicity effect against Raw 264.7 macrophage cells.

## 1. Introduction

Applications of conventional synthetic fungicides in crop protection have benefited farmers for decades. However, because of their prolonged applications, some crop pathogens have developed resistance against a wide spectrum of available fungicides [1,2,3]. Fungicide resistance is a major concern in agriculture, as it reduces pesticide efficacy and limits the number of pesticides available to treat or manage crop diseases. In addition to the resistance problem, there is public concern about pesticide residues in fruits and vegetables, as these chemicals are liable to remain in commodities following their applications. The residues pose serious health risks to consumers and have a negative impact on the environment, as well as on aquatic life [4,5,6,7,8]. Due to these challenges, researchers have focused on medicinal plants as an alternative source of compounds with the potential to be developed as new classes of fungicides. Plant-derived fungicides are less likely to negatively affect the environment, because they are naturally unstable at higher temperatures and light intensities; consequently, they may not persist in the environment for a very long time [7]. Medicinal plants are a source or reservoir of secondary metabolites such as flavonoids, alkaloids, steroids, terpenoids, tannins and other organic compounds with complex chemical structures. These secondary metabolites have various biological properties and can mediate chemical defense mechanisms in plants by creating barriers against pathogens and animals [7,9,10]. These active metabolites or compounds may be isolated, purified and characterized for industrial applications. Since natural compounds are present in very low quantities and are usually difficult to purify on a large industrial scale, the structure of such active compounds may be used as a template during the commercial/industrial production of pesticides [7].

As part of our search for antifungal compounds from medicinal plants against crop pathogens, in this study, we reported the antifungal activity of isolated compounds against selected *Fusarium* pathogens. The compounds were isolated from the leaves of *Combretum erythrophyllum* (Burch.) Sond. and *Withania somnifera* (L.) Dunal.

*Combretum erythrophyllum* belongs to the Combretaceae family, and it is commonly known as river bushwillow or bushveld willow. It is widely used in traditional medicine in Southern Africa [11,12]. *Withania somnifera* belongs to the Solanaceae family and is commonly known as ashwagandha, poison gooseberry, winter cherry or Indian ginseng [13]. It is used to treat various neurological disorders, diarrhea, gastrointestinal disorders, arthritis, stress and behavior-related problems [14,15]. *Withania somnifera* is also used as a nutrient and health restorative decoction by pregnant women and elderly people [16]. An infusion made from the leaves of this plant is used to treat fevers [17].

Some bioactive compounds have been isolated from *C. eryhtrophyllum* and *W. somnifera*, but to our knowledge, this is the first report describing the isolation and characterization, as well as the antifungal activity, of compounds from the leaves of these plants against *Fusarium* pathogens. Notwithstanding the promising antimicrobial activities demonstrated by medicinal plants against human and/or animal pathogens, there is a dearth of research related to the activity of plant extracts or compounds isolated from plants against crop pathogens. The aim of the present study was to evaluate the antifungal activity of the compounds isolated from *Combretum erythrophyllum* and *Withania somnifera* leaf extracts against *Fusarium* species pathogens. Extracts from these plant species were selected for isolation, because they demonstrated very strong antifungal activity, as reported previously in some in vitro studies [18,19].

## 2. Results

### 2.1. Antifungal Activity of Plant Extracts Using Thin Layer Chromatography-Bioautography Assay 

A bioautography assay of the *Combretum erythrophyllum* leaf acetone extract showed four active bands against *F. verticilloides*. There were at least two active bands recorded against *F. oxysporum*, *F. solani* and *F. graminearum.* However, some bands were located at similar distances and had the same retention factor (R_f_) (Table 1). The band with an R_f_ value of 0.47 strongly inhibited the growth of all seven pathogens tested. The *Withania somnifera* leaf ethyl acetate extract showed three active bands against *F. verticilloides*, *F. proliferatum* and *F. semitectum*. The bands with R_f_ values of 0.22 and 0.41 inhibited the growth of all the pathogens tested (Table 2). Figure S4 represents Thin Layer Chromatography Bioautography of *Combretum erythrophyllum* and *Withania somnifera* extracts against different *Fusarium* pathogens. 

### 2.2. Purified Compounds

The masses of the purified compounds obtained from the plant extracts after successive fractionation, precipitation or through TLC purification are presented in Table 3. The highest amount was obtained for compound **Z** with 2.7%. This compound was isolated from *W. somnifera* as a wax-like substance. The total percentage weights of the isolated compounds were 12.8% and 14.8% obtained from *C. erythrophyllum* and *W. somnifera*, respectively. 

### 2.3. Antifungal Activity of the Compounds 

Out of the 11 compounds isolated from *C. erythrophyllum* leaf acetone extract, five compounds showed strong antifungal activity (MIC < 1.0 mg/mL) against two or more of the tested pathogens (Table 4). Compound **A** exhibited very strong activity, with an MIC value of 0.01 mg/mL against *F. proliferatum*, and this is the strongest activity observed when compared to the other isolated compounds evaluated in this study. Compound **D** showed antifungal activity, with MIC values ranging between 0.3 and 0.63 mg/mL against all seven pathogens (Table 4). The antifungal activity of compound **D** against *F. oxysporum*, *F. subglutinans*, *F. solani*, *F. graminearum* and *F. chlamydosporum* was stronger than the activity demonstrated by the reference standard, amphotericin B^®^. On the other hand, out of the 12 compounds isolated from the *W. somnifera* leaf ethyl acetate extract, only three compounds showed strong activity against one or more of the tested pathogens (Table 5). The strongest antifungal activity was demonstrated by compound **AI** with an MIC value of 0.16 mg/mL against *F. verticilloides* (Table 5). In comparison to the reference standard (Amphotericin B^®^), this recorded activity was 53 times less effective against *F. verticilloides*.

### 2.4. Phytotoxicity of Isolated Antifungal Compounds on Maize Seed Germination

**Figure 1:**
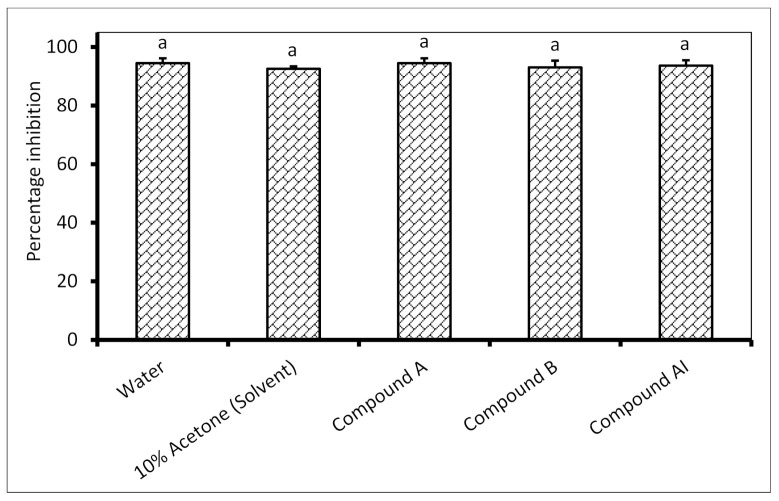
Percentage germination of maize seeds treated with isolated antifungal compounds. Compound **A** and compound **B** were isolated from the *Combretum erythrophyllum* leaf acetone extract. They were used at a concentration of 0.63 mg/mL in 10% acetone. Compound **AI** was isolated from the *Withania somnifera* leaf ethyl acetate extract, and its phytotoxicity was tested at a concentration of 0.16 mg/mL in 10% acetone. Water and 10% acetone were used as the negative controls. There were 5 replicates per treatment, each comprising 25 disinfected maize seeds, and the experiment was repeated twice. Data from the two repeat experiments were averaged and analyzed statistically. Bars bearing the same letters indicate no significant differences (*p* = 0.05), as determined by Duncan’s Multiple Range Test.

There is no significant difference in the maize seed germination of untreated seeds in comparison to all seeds treated with the isolated antifungal compounds (Figure 1). On average, the percentage seed germination for the control and all the treatments was above 93%.

### 2.5. Inhibition of Raw 264.7 Macrophage Cell Proliferation by Isolated Compounds

**Figure 2:**
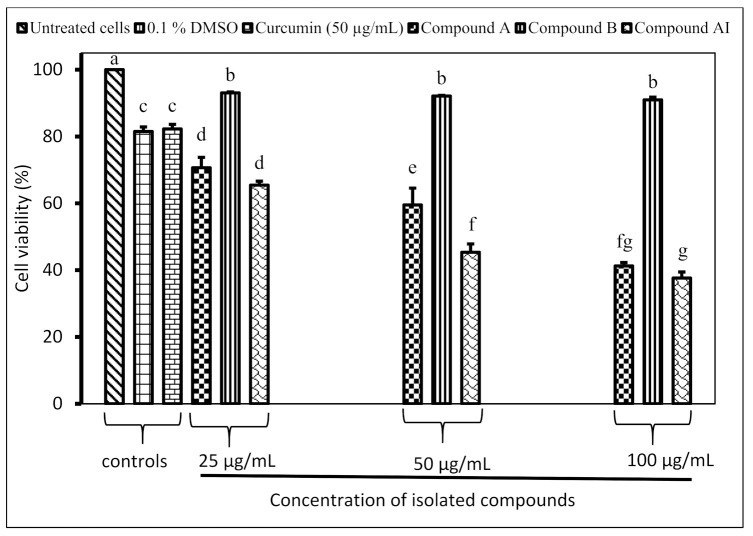
In vitro cytotoxicity of isolated compounds against the proliferation of Raw 264.7 macrophage cells. Compound **A** and compound **B** were isolated from the *Combretum erythrophyllum* leaf acetone extract. Compound **AI** was isolated from the *Withania somnifera* leaf ethyl acetate extract. A cell viability assay was determined using the MTT (3-(4,5-Dimethylthiazol-2-yl)-2,5-diphenyltetrazolium bromide) assay. Untreated cells, 0.1% DMSO and curcumin were included as controls. There were three replicates for each treatment group. Data were treated statistically, and bars bearing the different letters indicate significant differences between mean values (*p* = 0.05), as determined by Duncan’s Multiple Range Test.

Compound **A** and compound **AI** inhibited the proliferation of Raw 264.7 macrophage cells in a dose-dependent manner (Figure 2). The half-maximal inhibitory concentration (IC_50_) values were found to be 70.7 µg/mL and 48.2 µg/mL for compound **A** and compound **AI**, respectively. There are no significant differences in terms of the inhibition of compound **B** against the cell proliferation of Raw 264.7 at different tested concentrations (25, 50 and 100 µg/mL).

### 2.6. Structural Elucidation of Compounds

Compound **A** was isolated from the leaf acetone extract of *C. erythrophyllum* as a yellow powder. Its melting point was approximately 340–343 °C. The maximum absorbance of compound **A** was 270 nm, and there was a shoulder at 330 nm, which indicated the presence of a flavones skeleton or flavone derivative compound [20,21]. The positive electrospray ionization-mass spectrum (ESI-MS) of this compound showed peaks at the mass-to-charge ratio (*m*/*z*) of 284.9, 286.0, 327.0 and 328.0 and a molecular ion base peak at *m*/*z* 326.0. The proton nuclear magnetic resonance (^1^H-NMR) spectral data of compound **A** is presented in Table 6. The carbon 13 nuclear magnetic resonance (^13^C-NMR) spectral data of this compound is presented and compared with the literature data in Table 7. 

The ^1^H-NMR spectrum of compound **A** displayed the presence of four aromatic protons at δ_H_ 8.0 (2H, d, H-2′ and H-6′) and at δ_H_ 7.1 (2H, d, H-3′ and H-5′). This may suggest a substituted B ring of a flavones skeleton. The heteronuclear single quantum coherence (HSQC) spectrum confirmed a direct connection between the protons at δ_H_ 7.1 and 8.0 with the carbons at δ_C_ 115.9 and 128.6 ppm, respectively. This was further supported by two doublet signals at 7.1 and 8.0 ppm on the ^1^H-NMR spectrum. The proton–proton correlation spectroscopy (^1^H-^1^H COSY) spectrum indicates that these neighboring protons split each other (H-2′ is coupled with H-3′ and H-5′ with H-6′). There is also a single bond correlation of aromatic protons at δ_H_ 7.0, 6.8 and 6.5 with carbons at δ_C_ 103.8, 94.1 and 98.9, and they can be assigned to carbons 3, 8 and 6, respectively. The ^1^H-NMR spectrum showed overlapping peaks at δ_H_ 3.8 ppm, which can be assigned to three protons of two methoxy groups. The position of the methoxy protons was assigned to 3H, s, 4′-OMe, and the other one was assigned to 3H, s, 7-OMe. This was confirmed by ^13^C-NMR signals at 55.6 (C-4′) and 56.1 (C-7) and by the HSQC experiment, which showed a direct correlation of protons at δ_H_ 3.8 and 3.9 with carbons at δ_C_ 55.6 and 56.1, respectively.

**Figure 3:**
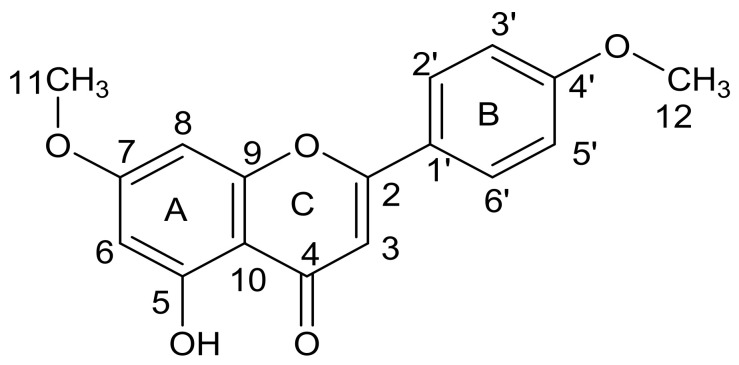
The structure of the flavonoid compound isolated from the *C. erythrophyllum* leaf acetone extract.

An examination of the ^13^C-NMR and distortionless enhancement by polarization transfer (DEPT, 135 °C) spectra revealed the presence of eight quaternary carbons at δ_C_ 103.5, 122.8, 157.4, 161.5, 162.3, 163.3, 164.3 and 181.8, which can be assigned to carbons 10, 1′, 9, 4′, 5, 7, 2 and 4, respectively. This is a characteristic of fused rings in flavone derivative compounds. The position of a hydroxyl group at carbon 5 was assigned based on the heteronuclear multiple bond correction (HMBC) long correlation of δ_H_ 12.9 (-OH) with carbons at δ_C_ 98.9 (C-6), 103.8 (C-3), 161.5 (C-4′), 162.3 (C-5), 163.3 (C-7) and 164.3 (C-2). Based on physical, spectroscopic data and comparison with the literature information, the structure of compound **A** was tentatively identified as 5-hydroxy-7,4′-dimethoxyflavone and is represented in Figure 3. Assignment of carbon atoms of compound **A** to chemical shift signals is indicated on ^13^C-NMR spectrum as presented in Figure S1.

Compound **B** was isolated from the *C. erythrophyllum* leaf acetone extract as an orange, oily substance that crystallized into a colorless material during evaporation in the fume cupboard. The melting point of this compound was 260–268 °C, and its maximum absorbance was 245 nm. The ^1^H-NMR spectral data of compound **B** is presented in Table 8, whilst its ^13^C-NMR data is presented and compared with the literature data in Table 9.

The ^13^C-NMR and ^1^H-NMR spectra of compound **B** displayed about twenty-six carbon signals and thirteen proton signals, respectively. A careful examination of the HSQC spectrum showed that the olefinic carbon signal at 124.3 ppm was connected directly to a proton resonating at 5.1 ppm. This proton signal (5.1 ppm) was split into a multiplet, presumably by neighboring protons. Another olefinic carbon signal was at 135.0 ppm and was displayed by the ^13^C-NMR and DEPT experiment as a quaternary carbon. Based on these assumptions or examinations, carbon signals resonating at 124.3 and 135.0 ppm may be assigned to C-12 and C-13, respectively. A clear examination of the ^1^H-^1^H COSY spectrum of compound **B** showed that protons at 1.9 ppm were in proximity with both protons at 2.1 and 5.1 ppm. Furthermore, all these proton signals (1.9, 2.1 and 5.1 ppm) appeared as triplets, and this may suggest that protons resonating at 1.9 ppm are attached to C-11, and the one at 2.1 ppm might be attached to C-9.

The DEPT spectrum of this compound showed about nine methylene carbon signals at 54.2, 39.7, 31.9, 29.6, 29.3, 28.2, 26.7, 26.6 and 22.6 ppm. These signals were assigned to carbons at C-1, 2, 6, 7, 15, 16, 22, 19 and 11. The number of methylene carbon signals may also suggest that the methyl carbons numbered 29 and 30 are connected to the same carbon. The HMBC experimental data showed a long-range connection between the proton at 2.2 ppm with the carbon signals at 207.1 and 44.8 ppm. This proton (2.2 ppm) was found to be connected to a carbon signal at 30.9 ppm, as evidenced by the HSQC experiment. Based on this information, the carbon signals resonating at 30.9 and 44.8 ppm may be assigned to C-5 and C-4, respectively. This assignment was also supported by the examination of both ^13^C-NMR and DEPT spectra, which showed a carbon signal at 44.8 ppm as the quaternary carbon. The ^13^C-NMR spectrum also showed the carbonyl signals at 207.1 and 178.1 ppm assignable to the ketone and carboxylic acid carbons numbered C-3 and C-28, respectively. 

**Figure 4:**
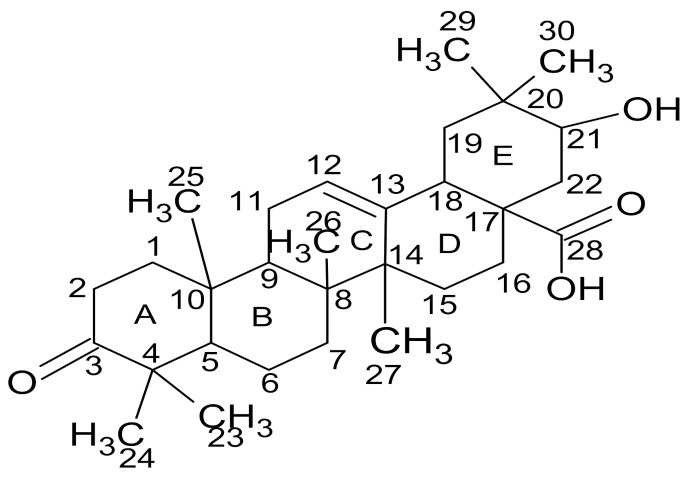
The structure of the triterpenes compound isolated from the *C. erythrophyllum* leaf acetone extract.

The ^1^H-NMR spectrum of compound **B** showed proton signals at the chemical shift ranging from 0.8 to 5.1 ppm. There are similarities between these chemical shifts obtained for compound **B** and the proton NMR data for 21β-hydroxyolean-12-en-3-one reported by Mena-Rejón et al. [24], who suggested the presence of a 12-oleanene type of triterpene molecule with a secondary hydroxyl and keto group. The positive mass fragmentation of compound **B** showed peaks at *m*/*z* 306.15, 400.25, 452.35, 482.35, 498.35, 516.35 and 530.35 and a molecular ion base peak at *m*/*z* 468.35. The molecular ion base peak is consistent with molecular formula C_30_H_44_O_4_ and is comparable with the data for 3-oxo-olean-12-en-28-oic acid synthesized by Wicht [25]. From these data, compound **B** was characterized as 21-hydroxy-3-oxo-olean-12-en-28-oic acid. The structure of the compound is presented in Figure 4 and is denoted as maslinic acid. Assignment of carbon atoms of compound **B** to chemical shift signals is indicated on ^13^C-NMR spectrum as presented in Figure S2.

Compound **AI** was isolated from the *W. somnifera* leaf ethyl acetate extract as a sticky yellowish substance. The positive mass fragmentation of compound **AI** indicated peaks at *m/z* 262.9, 399.1, 417.1, 435.1, 453.2, 488.0, 534.2, 942.6 and 958.5 and a molecular ion base peak at *m/z* 963.3. The proton NMR spectral data of this compound was compared with a withanolide derivative and withaferin A obtained from the literature in Table 10 [26,27]. The ^13^C-NMR data of compound **AI** was presented and compared with the withanolide derivative molecule and with withaferin A data in Table 11 [26,28].

**Figure 5:**
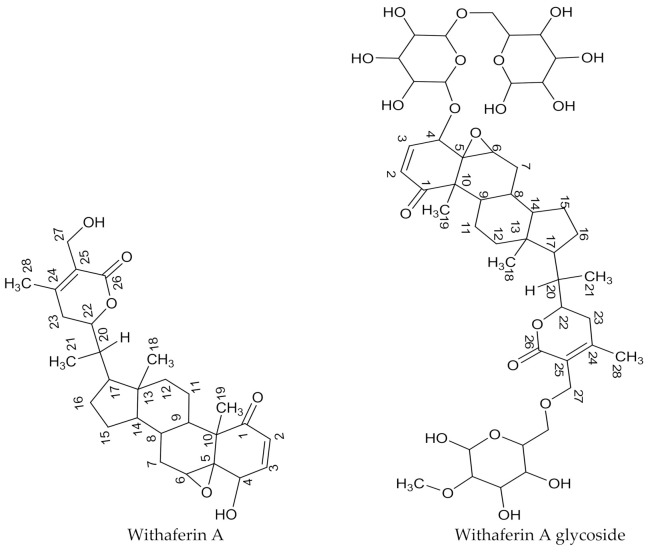
The structure of withaferin A and the withanolide glycoside compound isolated from the *W. somnifera* leaf ethyl acetate extract.

The ^13^C-NMR spectral analysis of compound **AI** showed 28 major carbon signals, and from DEPT experiment, seven of those are quaternary carbon signals. These quaternary carbons resonate at δ_C_ 203.2, 73.2, 57.2, 48.6, 150.6, 121.3 and 167.2 ppm and may be assigned to C-1, C-5, C-10, C-13, C-24, C-25 and C-26, respectively. The HSQC experiment further confirmed that there are no protons attached to these carbon atoms. The assignment of C-1 to the chemical shift signal appearing at δ_C_ 203.2 ppm corresponded very well with the ketone carbonyl resonance range. The carbon peak signal at 167.2 ppm was at the signal range characteristics of the carbonyl esters; hence, it was assigned to C-26. The ^13^C-NMR spectrum showed carbon signals at the chemical shifts 73.2 and 78.7 ppm. The type of carbon groups in this range are characteristics of carbon–oxygen linkage groups and may correspond to oxygen linkage to C-5 and C-22, respectively. The DEPT experiment further showed the presence of seven methylene groups at δ_C_ 21.6, 22.9, 32.4, 32.7, 36.5, 36.7 and 50.9 ppm, and these signals were assigned to C-7, C-11, C-12, C-15, C-15, C-23 and C-27. From these data, the structure of compound **AI** was characterized and identified as withaferin A (4β,27-dihydroxy-1-oxo-5β,6β-epoxywitha-2-24-dienolide). The identification of withaferin A was strongly supported by the number of quaternary carbon signals and carbon–oxygen linkage at C-5. However, a *m/z* cloud software search using mass spectroscopic data showed a withanolide glycoside compound. This suggests the presence of withaferin A glycoside in very small quantities; hence, no carbon signals from sugar moieties were detected during the NMR analysis. The structures of these compounds are presented in Figure 5. Assignment of carbon atoms of compound **AI** or withaferin A to chemical shift signals is indicated on ^13^C-NMR spectrum as presented in Figure S3.

## 3. Discussion

The bioautography determination of the antifungal profile of the extracts obtained from *C. erythrophyllum* and *W. somnifera* showed white bands at different R_f_ values. This suggests that the antifungal activity reported in the previous studies [18,19] was due to the combination of more than one compound. Regardless of the plant extracts and microorganisms tested, these observations are in agreement with other studies [29,30,31]. A bioautography assay helps to make informed decisions regarding the selection of extracts to be used during the fractionation and isolation of antifungal compounds. In the present study, *C. erythrophyllum* acetone and *W. somnifera* ethyl acetate extracts were selected due to the presence of active bands at R_f_ values 0.47 and 0.22 and 0.41, respectively. These active bands inhibited the growth of all the tested pathogens.

Compound **A** isolated from *C. erythrophyllum* demonstrated very strong antifungal activity with a MIC value of 0.01 mg/mL against *F. proliferatum*. This activity was four times stronger when compared to the MIC value of 0.04 mg/mL for the *C. erythrophyllum* crude acetone extract against *F. proliferatum* [19]. Medicinal plant extracts contain mixtures of different secondary metabolites, which may interact with each other to produce additive, synergistic or antagonistic antifungal effects. For that reason, the antifungal activity of an isolated compound may be completely different, and in this case, it was demonstrated by compound **A**. This compound was also found to be active (MIC value ranged from 0.31 to 0.63 mg/mL) against *F. verticilloides*, *F. solani*, *F. graminearum* and *F. chlamydosporum*; however, on the other hand, it was inactive (1.25–1.3 mg/mL) against *F. oxysporum* and *F. subglutinans*. Despite the microorganisms tested, other isolated compounds (compounds **B**, **I**, **J, AB, AF** and **AI**) also showed similar trends, and this suggests that the antifungal activity of the isolated compounds is pathogen-specific. It is noteworthy that compound **D** was active against all the tested pathogens, with MIC values less than 0.63 mg/mL. The antifungal activity demonstrated by compounds **B** and **D** was higher than that of the positive control (Amphotericin B^®^) used in this study against *F. oxysporum*, *F. subglutinans*, *F. solani* and *F. graminearum*. Compared to the positive control, compound **A** demonstrated a stronger inhibitory activity against *F. solani*, *F. graminearum* and *F. chlamydosporum*.

When compared to the negative control (water treatment), isolated compounds such as compounds **A**, **B** and **AI** showed no negative effects on maize seed germination. The phytotoxicity of the other isolated compounds was not determined due to the unavailability of sufficient material after several preliminary experiments. In a study by Saha et al., triterpenic saponins isolated from *Sapindus mukorossi* and *Diploknema butyracea* demonstrated growth-promoting activity on maize and rice seeds [32]. On the other hand, a purified extract obtained from the leaves of *Gleicheni linearis* was found to lower maize growth and yield when compared to crude extract and the control. This purified extract was found to contain kaempferol and other flavonoid compounds, presumably [33]. Plant-based products that do not negatively affect maize seed germination are of particular importance, since, in smallholder farming, surplus maize seeds are stored and used for planting in the next season [34]. Further studies are needed to evaluate the bioactivity of compounds **A**, **B** and **AI** on maize growth.

Notably, the current study revealed that compound **B** isolated from the *C. erythrophyllum* leaf acetone extract showed no severe toxicity against Raw 264.7 macrophage cells. The cell viability percentage of compound **B** was 92.1% and was significantly different from the 81.5% recorded for the positive control (curcumin) at a similar concentration. A dose-dependent cytotoxicity effect was evident for compounds **A** and **AI.** However, at concentrations of 25 µg/mL and 50 µg/mL, both compounds showed no severe toxicity against Raw 264.7 macrophage cells. The safety of these isolated compounds was also demonstrated by higher IC_50_ values of 1443.8, 70.7 and 48.2 µg/mL recorded for compounds **B**, **A** and **AI**, respectively. Although we evaluated the cytotoxicity against Raw 264.7 macrophage cells; according to the National Cancer Institute (NCI), Bethesda, MD, USA. Crude extracts and pure compounds can be considered as cytotoxic agents against cancerous cells if they exhibit IC_50_ values less than 20 µg/mL and 4 µg/mL, respectively [35].

Compounds **A** and **B** isolated from the *C. erythrophyllum* leaf extract and compound **AI** from the *W. somnifera* leaf extract were characterized to determine their names and structures. The structures of the other compounds isolated in this study were not determined due to a low quantity of available materials. The structure of compound **A** was characterized as 5-hydroxy-7,4′-dimethoxyflavone. This compound is likely to be apigenin-substituted with methoxy groups at positions 7 and 4′; however, it may have been formed as a breakdown of salvigenin, which lost a methoxy group at position 6. Apigenin (4′,5,7-trihydroxyflavone) is one of the most widely distributed flavonoids in the plant kingdom, and it belongs to the flavone subclass. This compound was isolated from different plant species such as *Chromolaena hirsute* and *Macaranga gigantifolia,* as well as from *Combretum erythrophyllum* [36,37,38]. Due to its nutritional and organoleptic properties, apigenin was included in different nutraceutical products or formulations [39]. Several studies have reported the antimicrobial activity of apigenin against Gram-positive and Gram-negative bacterial strains. As an example, apigenin was evaluated for antibacterial activity against different bacterial strains, such as *Staphylococcus aureus*, *Enterococcus faecalis*, *Escherichia coli*, *Pseudomonas aeruginosa*, *Salmonella typhimurium, Proteus mirabilis, Klebsiella pneumoniae, Enterobacter aerogenes* and *Streptococcus epidermidis* [40,41,42,43]. The antibacterial activity of apigenin reported in some of these studies was moderate to relatively low, with MIC values ranging from 500 µg/mL to 1000 µg/mL.

Salvigenin has been isolated from *Tanacetum canescens,* *Astragalus propinquus* and *Salvia officinalis* and was reported to exhibit antitumor activity and an analgesic effect [44,45,46]. To the best of our knowledge, the present study was the first to characterize 5-hydroxy-7,4′-dimethoxyflavone from the leaves of *Combretum erythrophyllum*. However, this same compound (5-hydroxy-7,4′-dimethoxyflavone) was isolated from *Combretum zeyheri*, which also belongs to the Combretaceae family [47]. In that study, 5-hydroxy-7,4′-dimethoxyflavone exhibited an antifungal activity with a MIC value of 45 µg/mL against *Candida albicans* [47]. The antimicrobial activity of 8-hydroxy-salvigenin was also reported with MIC values ranging from 0.098 mg/mL to 0.78 mg/mL against human and animal pathogens such as *Escherichia coli*, *Proteus vulgaris*, *Pseudomonas aeruginosa*, *Candida albicans*, *Candida glabrata*, *Candida guilliermondii*, *Candida parapsilosis* and *Candida krusei* [48].

More work is required to investigate the antimicrobial activity of apigenin and salvigenin, particularly against crop pathogens. These compounds may be evaluated individually or in combination to establish the nature of their interactions against pathogens. In the current study, compound **A**, characterized as 5-hydroxy-7,4′-dimethoxyflavone, which is closely related to apigenin and salvigenin, showed strong antifungal activity, with MIC values ranging from 0.01 mg/L to 0.63 mg/mL, against *F. verticilloides*, *F. proliferatum*, *F. solani*, *F. graminearum* and *F. chlamydosporum*. In addition to 5-hydroxy-7,4′-dimethoxyflavone, we isolated maslinic acid (compound **B**) from the leaves of *C. erythrophyllum*, and it showed very good antifungal activity (MIC values ranging from 0.08 mg/mL to 0.63 mg/mL) against six tested *Fusarium* pathogens. Maslinic acid is a naturally occurring pentacyclic triterpene, and it was first isolated or detected in *Crataegus oxyacantha* and, later, in several plant species, vegetables, herbs and fruits [49,50]. Maslinic acid was studied for health-promoting properties, such as antioxidant, antidiabetic, antitumor, antiviral, antibacterial and anti-inflammatory activities [50,51,52]. It exhibited good antibacterial activity against *Staphylococcus aureus*, methicillin-resistant *Staphylococcus aureus*, *Staphylococcus epidermidis*, *Streptococcus mutans, Enterococcus faecalis,* *Porphyromonas gingivalis*, *Fusobacterium nucleatum* and *Parvimonas micra* [52,53].

Compound **AI** isolated from *W. somnifera* demonstrated good antifungal activity, with a MIC value of 0.16 mg/mL against *F. verticilloides*. This compound was characterized as a withaferin A glycoside. Withaferin A was one of the first and important withanolide compounds isolated from *W. somnifera*. This group of compounds may occur in free form or as glycosides, and they exhibit good antibacterial and antifungal activities [54,55,56].

Many natural bioactive compounds are present in low concentrations and are difficult to purify at a large scale [7]. Nonetheless, the structures of the antifungal compounds (apigenin, salvigenin, maslinic acid and withaferin A glycoside) identified in the current study may be used to design a synthetic route of bioactive compounds, which can be produced in larger quantities in the laboratory for their application in crop protection. Additionally, analogs or derivatives of these antifungal compounds can be made by structural manipulation of the functional groups, which can then be tested for their biological activities. This study demonstrated that medicinal plants are a natural source of antifungal compounds that can be isolated and characterized. However, if the antifungal compounds are to be used in crop protection and commercial farming, further research is required to evaluate their toxicological properties and environmental effects, as well as the synthesis of the compounds and or their active derivatives in larger quantities.

## 4. Materials and Methods 

### 4.1. Collection of Plant Material and Preparation of the Extracts

Fresh leaves of *Combretum erythrophyllum* (Burch.) Sond. were collected from a naturally growing tree at the Agricultural Research Council, Roodeplaat, Gauteng Province, South Africa (S 25°36.206′ E 028°20.915′). *Withania somnifera* (L.) Dunal leaves were collected from Capricorn District, Limpopo Province, South Africa (E 23°47.745′ S 029°19.202′). Dr. Bronwyn Egan, Larry Leach Herbarium Curator at the University of Limpopo, authenticated the plants. Herbarium samples (Voucher numbers UNIN 121005 and UNIN 121010) for *Combretum erythrophyllum* and *Withania somnifera*, respectively, were deposited at the University of Limpopo Herbarium. 

The preparation and extraction of plant extracts were done as previously described [19]. In brief, green fresh leaves (approximately 10 Kg) were collected in brown paper bags and shade-dried at room temperature (25 ± 2 °C) immediately after collection. Dried leaf materials were ground (Fritsh Pulverisette 14, Labotec, Midrand, South Africa) into fine powder. *Combretum erythrophyllum* powdered material (300 g) was extracted with 3000-mL acetone, while *W. somnifera* material was extracted with ethyl acetate as the solvent. Each plant material was separately extracted on a shaker (Shaker LS500, Gerhardt, Analytical Systems, Bonn, Germany) for 1 h at room temperature. Extracts were filtered through Whatman No.1 filter paper, and the residues were re-extracted. The filtrates or plant extracts were concentrated under vacuum at 45 °C using rotary evaporators (Stuart, RE300DB, Lasec, Cape Town, South Africa). The concentrated extracts were further dried under a stream of air in a fume cupboard. The dried extracts were weighed, recorded and stored in airtight containers at room temperature.

### 4.2. Subculturing of Fusarium Pathogens

*Fusariumoxysporum* (PPRI 10175), *F. verticilloides* (PPRI 9278), *F. subglutinans* (PPRI 6740), *F. proliferatum* (PPRI 18679), *F. solani* (PPRI 19147), *F. graminearum* (PPRI 10728), *F. semitectum* (PPRI 6739) and *F. chlamydosporum* (PPRI 5116) were obtained from the Agricultural Research Council, Plant Health and Protection at Roodeplaat, South Africa. The pathogens were subcultured as previously described [19]. Briefly, the pathogens were cultured on potato dextrose agar and incubated at 27 °C in the dark for four days. Thereafter, the fungal suspension was scrapped off and inoculated into potato dextrose broth and incubated for four to seven days at 27 °C in the dark. The spores were collected through a cheesecloth and counted using a microscope (Zeiss Axioplan, Oberkochen, Germany) and a hemocytometer (Hirschmann, Eberstadt, Germany). The number of spores was determined and adjusted to 1.0 × 10^6^ spores/mL [57,58].

### 4.3. Thin Layer Chromatography (TLC) Bioautography

The extracts were reconstituted in acetone at 20 mg/mL. Thereafter, 20 µL was deposited as a concentrated spot on the TLC plate (aluminum-backed silica gel 60 F_254_, Merck, Darmstadt, Germany). The bottom edge of the plate was placed in a solvent tank (toluene:methanol:acetonitrile:acetic acid, 80:10:5:5, *v*/*v*). The solvent moved up the plate under capillary action, and when the solvent front reached the other edge of the plate, the plate was removed from the tank. It was placed under a stream of air in a fume cupboard for a week. The plate was sprayed with fine droplets of *Frium* pathogen (1.0 × 10^6^ spores/mL) in potato dextrose broth. It was incubated for two days and then sprayed with a solution of 2-mg/mL *p*-iodonitrotetrazolium (Sigma-Aldrich, Modderfontein, South Africa). The plate was incubated further overnight, and visualization of the antifungal bands was examined as white spots. Retention factor (R_f_) values of the white spots were recorded.

### 4.4. Compound Isolation

Open silica gel column chromatography (normal phase) was used to purify individual compounds from the extract. The column was packed with 100-g silica gel powder (Grade 7734, pore size 60 Å, 70-230 mesh, Sigma-Aldrich, Modderfontein, South Africa) into a glass tube to a height of 30 cm with a 3.5-cm internal diameter. Petroleum ether was added to the column and kept flowing for several minutes to equilibrate the column. The plant extract (3.0 g) dissolved completely in acetone was mixed with 15.0 g silica gel and dried in a stream of air in the fume cupboard. It was crushed into fine powder and loaded flat on top of the column. Cotton wool and filter paper cut to the size of the column were placed on top of the column to reduce the disturbances during the addition of the mobile phases.

Compound separation was initiated by adding 150 mL of 100% petroleum ether, followed by 50 mL of each of the following different solvent systems (*v*/*v*): petroleum ether:ethyl acetate (45:5), (35:15), (25:25), (15:35) and (5:45) and ethyl acetate:methanol (50:0), (45:5), (35:15), (25:25), (15:35) and (5:45). The column was allowed to run under gravity, and the fractions were collected into 20-mL glass pill vials (ChemLab Supplies, Johannesburg, South Africa). The column was washed with 150-mL absolute methanol and then with 200-mL dichloromethane. The fractions were concentrated using rotary evaporators (Stuart, RE300DB, Labotec, Midrand, South Africa). The fractions were checked for purity on a TLC plate through visualization using an ultraviolet lamp (Spectroline, ENF-240L/FE, Spectronics Corporation, Westbury, NY, USA). It was further checked by spraying with vanillin reagent (0.1-mg vanillin in 28-mL methanol and 1-mL concentrated sulphuric acid) and heated at 110 °C until color development. Fractions that showed similar TLC profiles were combined and further separated using a small column or TLC technique to obtain purified compounds. The compounds were weighed and kept in the vials. 

### 4.5. Antifungal Activity Determination of Isolated Compounds

The antifungal activity of the compounds was determined using the modified micro-plate dilution method [29]. Briefly, sterile potato dextrose broth (100 µL) was added to wells of a 96-well plate. Isolated compounds were dissolved in acetone at 10 mg/mL. The compound was added (100 µL) to the first well and serially diluted two-fold to the last well. Amphotericin B^®^ antibiotic (Phytotek Lab, Pretoria, South Africa) was included as a positive control, while different acetone strengths, sterile potato dextrose broth and water were the negative controls. One hundred microliters of pathogen subcultured in potato dextrose broth adjusted to 1.0 × 10^6^ spores/mL was added to the relevant wells. The plate was sealed and incubated for two to three days at 27 °C. Thereafter, 50 µL of 0.5-mg/mL *p*-iodonitro-tetrazolium violet was added to all wells and incubated overnight. Minimum inhibitory concentration (MIC) values were recorded as the lowest concentrations of the compounds that inhibited the growth of the pathogen, as indicated by no color changes (colorless) after incubation in the presence of growth indicators [59]. The assay was repeated twice in triplicate. For convenience, the isolated compounds were labeled alphabetically.

### 4.6. Physical and Spectroscopic Identification of Purified Compounds

HPLC grade acetonitrile and LC water were purchased from Lab-scan Analytical Sciences, Gliwice, Poland and from Macron Fine Chemicals, Avantor, Poland, respectively. Ammonium formate and formic acid were obtained from Sigma Aldrich, Modderfontein, South Africa. Melting points of the compounds were obtained using a melting point apparatus (Stuart SMP3, Barloworld Scientific, Stone, Staffordshire, UK). Ultraviolet-visible spectra were recorded using a spectrophotometer (Specord 210, Analytic Jena AG, Jena, Germany) and liquid chromatography-photodiode array detector (LC-PDA, Shimadzu Scientific Instruments, Tokyo, Japan). 

Mass spectra were recorded using liquid chromatography-mass spectrometer (LC-MS-2020, Shimadzu Scientific Instruments, Tokyo, Japan) at the Agricultural Research Council-Vegetables, Industrial and Medicinal Plants Analytical Laboratory, Pretoria, South Africa. The instrument had an electrospray ionization (ESI) source operating in negative and positive modes (*m*/*z* 100–1200), nebulizing gas (1.5 ℓ/min), DL temperature (250 °C), heat block temperature (200 °C) and detector voltage (0.19 Kv). Reserpine and nitrophenol were used as commercial compounds to calibrate and tune the mass spectrophotometer. The chromatographic system consists of a reverse-phase shim-pack C18 column (5 µm, 250 mm × 2.1 internal diameter) and a mixture of A (10-mM ammonium formate in 90% acetonitrile:water, *v*/*v*) and mixture B (0.1% formic acid in acetonitrile, *v*/*v*) as the mobile phases. Data acquisition was executed using LabSolution software and recorded as the absolute intensity (*m*/*z* values). MS data was exported and loaded into *m*/*z* cloud software to search compounds matching the MS fingerprint [60]. 

Nuclear Magnetic Resonance (NMR) (Ascend 400 MHz Topspin 3.2, Bruker, Billerica, MA, USA): ^1^H NMR and ^13^C NMR spectra were referenced internally using solvent signals. ^1^H NMR: 7.250 ppm for CDCl_3_ and 2.500 ppm for DMSO-d_6_; ^13^C NMR: 77.00 ppm for CDCl_3_ and 39.40 ppm for DMSO-d_6_, respectively, which were used as the solvents at room temperature. Chemical shifts are expressed as δ-values in parts per million (ppm) and the coupling constants (*J*) in Hertz (Hz). The multiplicity of the signals is given as follows: brs = broad singlet, s = singlet, d = doublet, dd = doublet of doublet and m = multiplet. For convenience, the atoms or signals were numbered numerically; however, the numbering system may not reflect the nomenclature systematic numbering of the compounds.

### 4.7. Characterization of Isolated Purified Compounds

Compound **A** was obtained as a yellow powder; m.p = 340–343 °C; UV (MeOH): λmax = 270 nm and 330 nm; ^1^H NMR (400 MHz, DMSO-d_6_): 3.8 (s, 3H), 6.2 (d, 1H, *J* = 2,04), 6.5 (d, 1H, *J* = 2.04), 6.8 (s, 1H), 7.1 (d, 2H, *J* = 8.96), 8.0 (d, 2H, *J* = 8.92), 10.9 (s, 1H), 12.9 (s, 1H); ^13^C-NMR (100 MHz, DMSO-d_6_): 55.6, 56.1, 94.1, 98.9, 103.6, 103.8, 115.9, 122.8, 128.6, 157.4, 161.5, 162.3, 163.3, 164.3, 181.8 ppm; LC-MS (ESI): *mz* = 326.0 [M + H]^+^. 

Compound **B** was obtained as colorless crystals upon evaporation; m.p = 260–268 °C; UV (MeOH or EtOH): λmax = 245 nm; ^1^H NMR (400 MHz, CDCl_3_): 0.8 (s, 3H), 0.9 (t, 2H, *J* = 6.73 Hz), 1.0 (s, 3H), 1.3 (s, 3H), 1.7 (s, 1H), 1.8 (s, 2H), 1.9 (d, 1H, *J* = 7.08 Hz), 2.0 (dd, 2H, *J* = 6.56 Hz), 2.1 (d, 1H, *J* = 6.88 Hz), 2.2 (s, 2H), 3.5 (s, 3H), 3.6 (s, 2H), 5.1 (td, 1H, *J* = 7.42 Hz, 14.4 Hz); ^13^C-NMR (100 MHz, CDCl_3_): 15.9, 16.3, 17.6, 18.6, 22.6, 25.7, 26.6, 26.7, 28.2, 29.3, 29.6, 30.9, 31.9, 39.7, 44.4, 44.8, 51.8, 54.2, 124.2, 124.3, 131.2, 135.0, 176.9, 178.1, 207.1 ppm; LC-MS (ESI): *mz* = 468.35 [M + H]^+^.

Compound **AI** was obtained as a sticky yellowish substance; ^1^H NMR (400 MHz, CDCl_3_): 0.8 (s, 2H), 0.9 (s, 1H), 0.9 (d, 1H, *J* = 8 Hz), 1.0 (d, 2H, *J* = 6.8 Hz), 1.1 (s, 1H), 1.2 (s, 1H), 1.3 (m, 3H, *J* = 6.4 Hz), 1.5 (m, 2H, *J* = 3 Hz, 12 Hz), 1.6 (m), (1.7 (m, 3H), 1.8 (s), 1.9 (s, 2H), 2.0 (m, 2H), 2.3 (m, 1H, *J* = 4 Hz), 2.4 (d, 1H, *J* = 3.6), 2.5 (m, 1H), 2.6 (d, 1H, *J* = 4.8 Hz), 2.7 (d, 1H, *J* = 18 Hz ), 2.8 (dd, 1H, *J* = 3 Hz, 13.4 Hz), 3.0 (d, 1H, *J* = 2.24 Hz), 3.1 (d, 1H, *J* = 1.20 Hz), 3.3 (m, 1H, *J* = 1.96 Hz, 5.44 Hz), 4.6 (m, 1H, *J* = 2.84 Hz, 7.36 Hz, 19.32 Hz), 5.8 (dd, 1H, *J* = 2.32 Hz, 10.28 Hz), 6.6 (m, 1H, 2.36 Hz, 7.44 Hz, 17.48 Hz); ^13^C-NMR (100 MHz, CDCl_3_): 9.5, 12.3, 14.7, 15.1, 20.5, 21.6, 22.9, 32.4, 32.7, 35.2, 35.9, 36.5, 36.7, 42.8, 45.8, 48.6, 50.9, 56.2, 57.2, 73.2, 78.7, 84.6, 121.3, 128.9, 139.7, 150.6, 167.2, 203.2 ppm; LC-MS (ESI): *mz* = 963.3 [M + H]^+^.

### 4.8. Phytotoxicity Evaluation of Isolated Antifungal Compounds against Maize Seed Germination

Characterized isolated antifungal compounds were evaluated for possible phytotoxicity on maize seed germination using the method described by Seepe et al. [61]. Briefly, seeds were soaked in a mixture of apigenin and salvigenin, as well as in maslinic acid, at a predetermined concentration of 0.63 mg/mL in 10% acetone. Another set of seeds were soaked in withaferin A glycoside prepared at a concentration of 0.16 mg/mL and air-dried in a biosafety cabinet for 1 h. Water and 10% acetone were used as controls. Twenty-five seeds were placed per petri dish lined with a moistened double layer of filter paper. Each treatment was replicated five times. The experiment was kept in an incubator at a constant 25 °C and an alternating cycle of 12-h light and 12-h darkness. The filter papers were kept wet throughout the experiment. The number of germinated seeds was recorded 3 days after sowing. The experiment was repeated twice. Percentage seed germination was calculated using the following Equation (1):

(1) $$\mathrm{Percentage}\;\mathrm{seed}\;\mathrm{germination}=\left(\frac{\mathrm{Number}\;\mathrm{of}\;\mathrm{germinated}\;\mathrm{seeds}}{\mathrm{Total}\;\mathrm{number}\;\mathrm{of}\;\mathrm{seeds}}\right)\times 100$$

### 4.9. Cytotoxicity Evaluation of Isolated Compounds

Raw 264.7 macrophage cell were cultured on Dulbecco’s Modified Eagle’s Medium (DMEM) supplemented with 10% inactivated Fetal Bovine Serum (FBS); 2-mM L-glutamine and a mixture of 10X (PSN), penicillin (100 IU/mL), streptomycin (100 µg/mL) and neomycin (5 µg/mL). They were maintained at 37 °C in a humidified 95% air and 5% CO_2_ environment using a carbon dioxide incubator (Thermo Fisher Scientific, Waltham, MA, USA). Confluence was allowed to take place, and the cells were dissociated and harvested using 0.2% trypsin, 0.02% EDTA and 0.05% glucosein Phosphate-Buffered Saline (PBS) solution. Raw 264.7 macrophage cells were seeded at a density of 6 × 10^4^ per well in a 96-well plate and incubated at 37 °C in a CO_2_ incubator overnight.

Cell viability was determined using the MTT (3-(4,5-dimethylthiazol-2-yl)-2,5-diphenyltetrazolium Bromide) assay [62]. In brief, 100 µL of monolayer cell culture adjusted to 1.0 × 10^5^ cells/mL using DMEM (Hyclone, Logan, BOS, USA) containing 10% FBS (Sigma-Aldrich, Darmstadt, Germany) was added to each well of the 96-well microtiter plates and incubated overnight at 37 °C in a CO_2_ incubator. After 24 h, the cells were then treated with each purified isolated compound (compound **A**, compound **B** and compound **AI**) at various concentrations (25, 50 and 100 µg/mL); 0.1% DMSO in culture medium and 50-µg/mL curcumin (Sigma-Aldrich, Darmstadt, Germany) for 24 h. Control cells were supplemented only with a medium containing 2% FBS. The plates were incubated at 37 °C for 3 days in a 5% CO_2_ environment, and observations were carried out at every 24-h interval.

After three days, all solutions in the wells were discarded, the cells were washed with PBS and 10 µL of MTT reagent (5 mg/mL, Sigma Aldrich, St. Louis, MO, USA) were added into each well and then incubated for 3 h at 37 °C in a CO_2_ incubator. The formed blue formazan crystals were dissolved in 200-µL DMSO (Saarchem, Honeydew, South Africa) by incubating in the dark for 30 min. Absorbance was measured at 560 nm using a GloMax-Multi microplate reader (Promega, Madison, WI, USA). The relative cell viability or percentage growth inhibition was calculated using the following Equation (2):

(2) $$\mathrm{Cell}\;\mathrm{viability}\;\mathrm{or}\;\mathrm{percentage}\;\mathrm{inhibition}=\left(\frac{\mathrm{Absorbance}\;\mathrm{of}\;\mathrm{the}\;\mathrm{sample}/\mathrm{treated}}{\mathrm{Absorbance}\;\mathrm{of}\;\mathrm{the}\;\mathrm{control}/\mathrm{untreated}}\right)\times 100$$

The concentration of the isolated compound required to inhibit cell growth by 50% (IC_50_ value) was generated by transforming, normalizing and fitting the absorbance and concentration values on a dose-response curve using GraphPad Prism version 5.0. Data were generated from three experiments, and each experiment was performed in triplicate.

### 4.10. Statistical Analysis

Phytotoxicity and cytotoxicity data was analyzed using STATISTICA-8 software. The differences between the treatments for each parameter were evaluated using one-way analysis of variance (ANOVA). Data were expressed as the mean ± standard error. Where a statistical significance (*p* = 0.05) was established, means separation was done using Duncan’s Multiple Range Test (DMRT).

## 5. Conclusions

In this study, the compounds characterized as 5-hydroxy-7,4′-dimethoxyflavone and maslinic acid (21-hydroxy-3-oxo-olean-12-en-28-oic acid) were isolated from the leaves of *C. erythrophyllum*, while withaferin A (4β,27-dihydroxy-1-oxo-5β,6β-epoxywitha-2-24-dienolide) was isolated from the leaves of *W. somnifera*. The structures of these compounds were established based on the physical properties and spectroscopic and literature data. The results from the in vitro experiments showed that these compounds possess antifungal activity against the tested *Fusarium* pathogens, and they have no negative effect on maize seed germination. Moreover, these compounds were not severely toxic to Raw 264.7 macrophage cells. The compounds isolated in this study may serve as potential biopesticides for the treatment of diseases in crop production and postharvest storage.

## Figures and Tables

**Table 1:** R_f_ values of antifungal bands in *Combretum erythrophyllum* leaf extracts using the TLC bioautography assay.

Pathogens	Leaf Extracts									
	Ethyl Acetate						Acetone			
*F. oxysporum*	-	-	-	-	-	-	-	-	0.47	0.52
*F. verticilloides*	0.20	0.29	0.38	0.44	0.48	-	0.32	0.38	0.47	0.52
*F. subglutinans*	-	-	0.38	-	0.48	0.51	-	0.38	0.47	0.52
*F. proliferatum*	0.20	-	0.38	0.44	0.48	-	-	0.38	0.47	0.52
*F. solani*	-	0.29	-	0.44	0.48	-	-	0.87	0.47	-
*F. graminearum*	-	-	-	-	-	-	-	0.38	0.47	-
*F. chlamydosporum*	-	-	0.38	0.44	0.48	0.51	0.32	-	0.47	0.52

**Table 2:** R_f_ values of antifungal bands in *Withania somnifera* leaf extracts using the TLC bioautography assay.

Pathogens	Leaf Extracts						
	Ethyl Acetate			Acetone			
*F. oxysporum*	0.22	0.41	-	0.26	-	0.44	-
*F. verticilloides*	0.22	0.41	0.59	0.26	-	-	-
*F. proliferatum*	0.22	0.41	0.59	0.26	0.33	-	-
*F. semitectum*	0.22	0.41	0.59	0.26	-	0.44	0.46
*F. solani*	0.22	0.41	-	0.26	0.33	-	-

**Table 3:** Masses of the compounds (*w*/*w*) isolated from leaf extracts of *Combretum erythrophyllum* and *Withania somnifera*.

*Combretum erythrophyllum*		*Withania somnifera*	
Compounds	Mass (*w*/*w* %)	Compounds	Mass (*w*/*w* %)
**A**	1.6	**Y**	1.4
**B**	1.0	**Z**	2.7
**C**	1.7	**AA**	1.3
**D**	1.1	**AB**	1.1
**E**	1.2	**AC**	1.0
**F**	0.9	**AD**	1.1
**G**	1.3	**AE**	0.2
**H**	0.7	**AF**	1.1
**I**	1.1	**AG**	0.8
**J**	0.9	**AH**	1.3
**K**	1.3	**AI**	1.4
		**AJ**	1.3

**Table 4:** Minimum inhibitory concentration (MIC) values of the compounds isolated from *Combretum erythrophyllum* leaf acetone extract and investigated for antifungal activity against phytopathogenic fungi.

Compounds	MIC (mg/mL)						
	*F. oxysporum*	*F. verticilloides*	*F. subglutinans*	*F. proliferatum*	*F. solani*	*F. graminearum*	*F. chlamydosporum*
**A**	1.25	**0.31**	1.3	**0.01**	**0.31**	**0.63**	**0.63**
**B**	**0.31**	**0.08**	**0.63**	**0.31**	**0.63**	**0.63**	1.3
**C**	>2.5	2.5	1.25	>2.5	>2.5	1.3	2.5
**D**	**0.63**	**0.63**	**0.63**	**0.63**	**0.63**	**0.63**	**0.3**
**E**	>2.5	>2.5	>2.5	>2.5	>2.5	>2.5	>2.5
**F**	2.5	>2.5	>2.5	>2.5	>2.5	2.5	1.3
**G**	2.5	>2.5	>2.5	>2.5	> 2.5	2.5	>2.5
**H**	1.25	>2.5	1.25	>2.5	1.3	1.3	1.3
**I**	**0.63**	>2.5	**0.63**	>2.5	1.3	2.5	>2.5
**J**	**0.63**	**0.63**	**0.63**	1.3	1.3	1.3	**0.63**
**K**	1.3	1.3	2.5	1.3	1.3	1.3	1.3
Amphotericin B^®^	1.2	0.003	9.4	0.0004	1.2	2.3	2.3

Values highlighted in bold indicate antifungal activity with MIC less than 1.0 mg/mL.

**Table 5:** Minimum inhibitory concentration (MIC) values of the compounds isolated from *Withania somnifera* leaf ethyl acetate extract investigated for antifungal activity against phytopathogenic fungi.

Compounds	MIC (mg/mL)				
	*F. oxysporum*	*F. verticilloides*	*F. proliferatum*	*F. semitectum*	*F. solani*
**Y**	>2.5	>2.5	>2.5	>2.5	>2.5
**Z**	>2.5	>2.5	>2.5	>2.5	>2.5
**AA**	>2.5	>2.5	>2.5	>2.5	>2.5
**AB**	**0.63**	**0.31**	**0.31**	1.25	1.25
**AC**	2.5	>2.5	2.5	2.5	2.5
**AD**	2.5	1.25	2.5	>2.5	2.5
**AE**	2.5	1.25	2.5	2.5	1.25
**AF**	>2.5	**0.63**	>2.5	**0.63**	**0.63**
**AG**	>2.5	>2.5	1.25	>2.5	>2.5
**AH**	>2.5	>2.5	2.5	>2.5	>2.5
**AI**	1.25	**0.16**	1.25	1.25	2.5
**AJ**	>2.5	>2.5	2.5	>2.5	>2.5
Amphotericin B^®^	1.2	0.003	0.0004	2.3	1.2

Values highlighted in bold indicate antifungal activity with MIC less than 1.0 mg/mL.

**Table 6:** ^1^H-NMR spectral data of compound **A** isolated from the *C. erythrophyllum* leaf acetone extract.

Signals	Chemical Shift (δ_H,_ ppm in DMSO-d_6_)	Integration, Multiplicity	Coupling Constant (*J*, Hz)	Apigenin	Salvigenin
1	3.8	s, 3H	-	-	3.87 (s, 3H)
2	6.2	d, 1H	2.04	6.19 (d, 1H, *J* = 2.0 Hz)	-
3	6.5	d, 1H	2.04	6.48 (d, 1H, *J* = 2.0 Hz)	6.52 (s, 1H)
4	6.8	s, 1H	-	6.78 (s, 1H)	-
5	7.1	d, 2H	8.96	6.94 (d, 2H, *J* = 8.8 Hz)	6.99 (d, 2H, *J* = 8.9 Hz)
6	8.0	d, 2H	8.92	7.94 (d, 2H, *J* = 8.8 Hz)	7.82 (d, 2H, *J* = 8.9 Hz)
7	10.9	s, 1H	-	-	-
8	12.9	s, 1H	-	12.97 (s, 1H)	12.74 (s, 1H)

**Table 7:** ^13^C-NMR spectral data of compound **A** showing similarities with the literature data [22].

Signals	Chemical Shift (δ_C_, ppm in DMSO-d_6_)	
	Compound A	Apigenin [22]
1	55.6	-
2	56.1	-
3	94.1	93.9
4	98.9	98.9
5	103.5	102.8
6	103.8	103.7
7	115.9	115.9
8	122.8	121.2
9	128.6	128.5
10	157.4	157.3
11	161.5	161.2
12	162.3	161.5
13	163.3	163.7
14	164.3	164.3
15	181.8	181.8

**Table 8:** ^1^H-NMR spectral data of compound **B** isolated from the *C. erythrophyllum* leaf acetone extract.

Signals	Chemical Shift (δ_H,_ ppm in CDCl_3_)	Integration, Multiplicity	Coupling Constants (*J*, Hz)
1	0.8	s, 3H	-
2	0.9	t, 2H	6.75
3	1.0	s, 3H	-
4	1.3	s, 3H	-
5	1.7	s, 1H	-
6	1.8	s, 2H	-
7	1.9	d, 1H	7.08
8	2.0	dd, 2H	6.56
9	2.1	d, 1H	6.88
10	2.2	s, 2H	-
11	3.5	s, 7H	-
12	3.6	s, 2H	-
13	5.1	td, 1H	7.42, 14.4

**Table 9:** ^13^C-NMR spectral data of compound **B** showing similarities with the reported literature data [23].

Signals	^13^C, ppm in CDCl_3_	
	Compound B	Ursolic Acid [23]
1	15.9	16.1
2	16.3	16.9
3	17.6	17.8
4	18.6	18.9
5	22.6	21.9
6	25.7	24.1
7	26.6	24.7
8	26.7	27.8
9	28.2	28.4
10	29.3	29.1
11	29.6	29.1
12	30.9	31.1
13	31.9	33.6
14	39.7	39.3
15	39.7	39.4
16	44.4	42.5
17	44.8	47.7
18	51.8	47.9
19	54.2	53.2
20	124.2	-
21	124.3	125.4
22	131.2	-
23	135.0	139.0
24	176.9	-
25	178.1	179.1
26	207.1	-

**Table 10:** ^1^H-NMR chemical shifts of compound **AI** showing similarities with the withanolide derivative data [26,27].

Signals	Compound AI			Withanolide Derivative [26]	Withaferin-A [27]
	Chemical Shift (δ_H,_ ppm in CDCl_3_)	Multiplicity	Coupling Constants (*J*, Hz)	δ_H,_ ppm	
1	0.8	s, 2H	-	0.89, m	-
2	0.9	s, 1H	-	0.94, m	0.91, s
3	0.9	d, 1H	8	0.96, m	-
4	1.0	d, 2 H	6.8	1.03, m	1.03, d
5	1.1	s, 1 H		-	-
6	1.2	s, 1H		1.20, m	1.20, s
7	1.3	m, 3H	6.4	-	1.27–1.34, m
8	1.5	m, 2H	3, 12	1.40, dd,	-
9	1.6	m		1.62, td	1.64–1.65, m
10	1.7	m, 3H		1.74, m	1.68–1.91, m
11	1.8	s		1.81–1.85, m	-
12	1.9	s, 2H		1.90, m	1.91, s
13	2.0	m, 2H		2.01, dd	2.0. s
14	2.3	m, 1H	4	2.32, dd	-
15	2.4	d, 1H	3.6	-	-
16	2.5	m, 1H	-	-	2.52–2.57, m
17	2.6	d, 1H	4.8	-	-
18	2.7	d, 1H	18	-	2.68–2.73, m
19	2.8	dd, 1H	3, 13.44	2.88, dd	-
20	3.03	d, 1H	2.24	2.93, dd	3.06, d
21	3.1	d, 1H	1.20	-	-
22	3.3	m, 1H	1.96, 5.44	-	-
23	4.6	m, 1H	2.84, 7.36, 19.32	4.55, m	4.30–4.41, m
24	5.8	dd, 1H	2.32, 10.28	4.83, d	5.86, dd
25	6.6	m, 1H	2.36, 7.44, 17.48	-	6.59–6.62, m

**Table 11:** ^13^C-NMR chemical shifts of compound **AI** showing similarities with the reported literature data [26,28].

Signals	^13^C, ppm in CDCl_3_		
	Compound AI	Withanolide Derivative [26]	Withaferin A [28]
1	9.5	-	9.8
2	12.3	11.6	11.6
3	14.7	13.4	13.3
4	15.1	16.0	17.4
5	20.5	20.0	20.0
6	21.6	21.5	22.2
7	22.9	24.3	24.3
8	32.4	31.3	29.8
9	32.7	32.9	31.2
10	35.2	-	-
11	35.9	-	-
12	36.5	39.0	38.8
13	36.7	39.6	39.4
14	42.8	42.5	42.6
15	45.8	42.7	44.2
16	48.6	-	-
17	50.9	51.7	51.9
18	56.2	56.1	56.1
19	57.2	58.0	57.4
20	73.2	74.2	69.9
21	78.7	77.9	78.8
22	84.6	78.4	80.0
23	121.3	-	125.1
24	128.9	127.2	131.6
25	139.7	-	137.5
26	150.6	154.1	152.6
27	167.2	166.4	166.9
28	203.2	210.1	202.2

## Data Availability

On request, the computational data is available from W.N.

## Supplementary Materials

The following are available online. Figure S1: 13C-NMR spectrum of flavonoid compound isolated from *C. erythrophyllum* leaf acetone extract, Figure S2: 13C-NMR spectrum of compound B showing similarities with reported literature data, Figure S3: 13C-NMR spectrum of withanolide compound isolated from *W. somnifera* leaf ethyl acetate extract, Figure S4: Thin Layer Chromatography (TLC) Bioautography of *Combretum erythrophyllum* and *Withania somnifera* extracts treated with different Fusarium pathogens. (A): Antifungal activity of *C. erythrophyllum* and *W. somnifera* acetone extracts against *F. verticilloides*. (B): Antifungal activity of *C. erythrophyllum* and *W. somnifera* ethyl acetate extracts against *F. proliferatum*. (C): Antifungal activity of *C. erythrophyllum* and *W. somnifera* ethyl acetate extracts against *F. solani.* Amphotericin B was included as positive control.

## Sample Availability

Samples of the compounds **A**, **B** and **AI** are available from the authors.
