# Supplementary material for: Antifungal Activity of Isolated Compounds from the Leaves of Combretum erythrophyllum (Burch.) Sond. and Withania somnifera (L.) Dunal against Fusarium Pathogens
Source: Molecules. 2021 Aug 5;26(16):4732. doi: 10.3390/molecules26164732 (PMC8401019; doi:10.3390/molecules26164732)
Supplement: Supplementary file 1 [file molecules-26-04732-s001.zip › molecules-1305449-supplementary.pdf]

molecules

MDPI

Article

Antifungal activity of isolated compounds from the leaves of *Combretum erythrophyllum* (Burch.) Sond. and *Withania somnifera* (L.) Dunal against *Fusarium* pathogens

Hlabana A. Seepe[1,2] *, Tselane G. Ramakadi[2], Charity M. Lebepe[2], Stephen O. Amoo[1,3,4] and Winston Nxumalo[2] *

1 Agricultural Research Council—Vegetables, Industrial and Medicinal Plants, Roodeplaat, Private Bag X293, Pretoria 0001, South Africa
2 Department of Chemistry, University of Limpopo, Private Bag X1106, Sovenga, 0727, Polokwane, South Africa
3 Indigenous Knowledge Systems Centre, Faculty of Natural and Agricultural Sciences, North-West University, Private Bag X2046, Mmabatho 2735, South Africa
4 Department of Botany and Plant Biotechnology, Faculty of Science, University of Johannesburg, P.O. Box 524, Auckland Park 2006, South Africa

* Correspondence: seepeh@arc.agric.za; Tel.: +27 12 808 8000 (H.A.S.); winston.nxumalo@ul.ac.za; Tel.: +27 015 268 2331 (W.N)

Received: 2 July 2021; Accepted: 30 July 2021; Published: 4 August 2021

Supplementary Materials

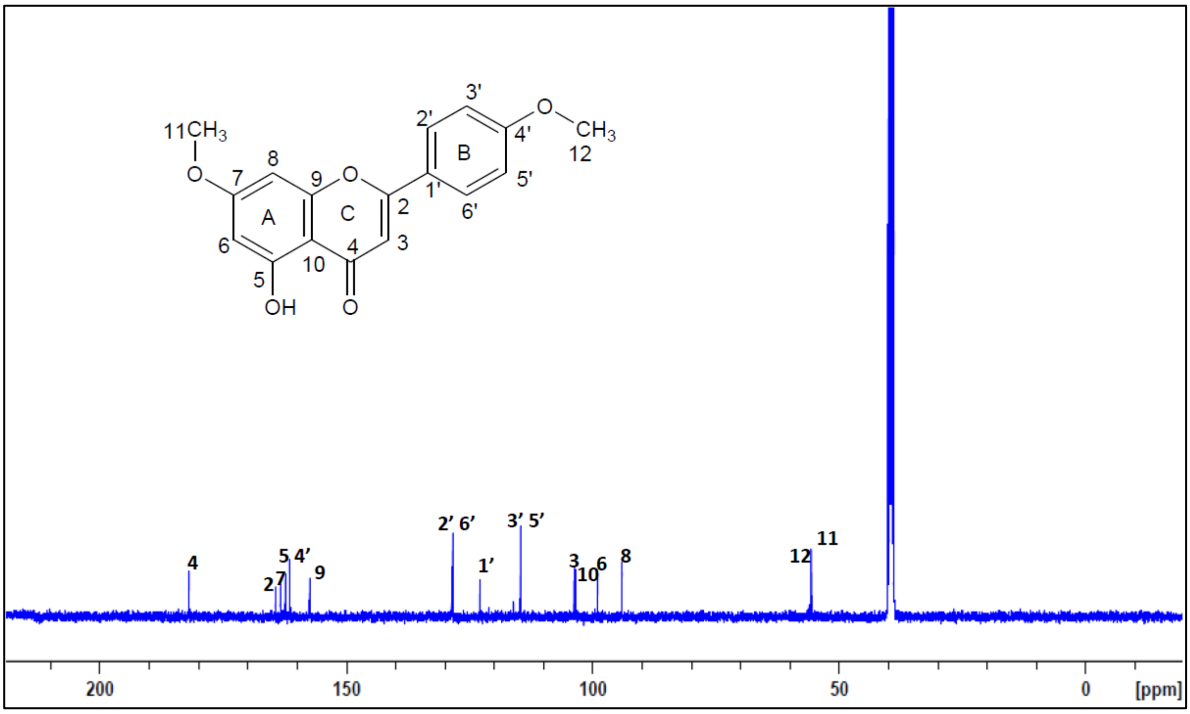

Figure S1. ^{13}C-NMR spectrum of flavonoid compound isolated from *C. erythrophyllum* leaf acetone extract.

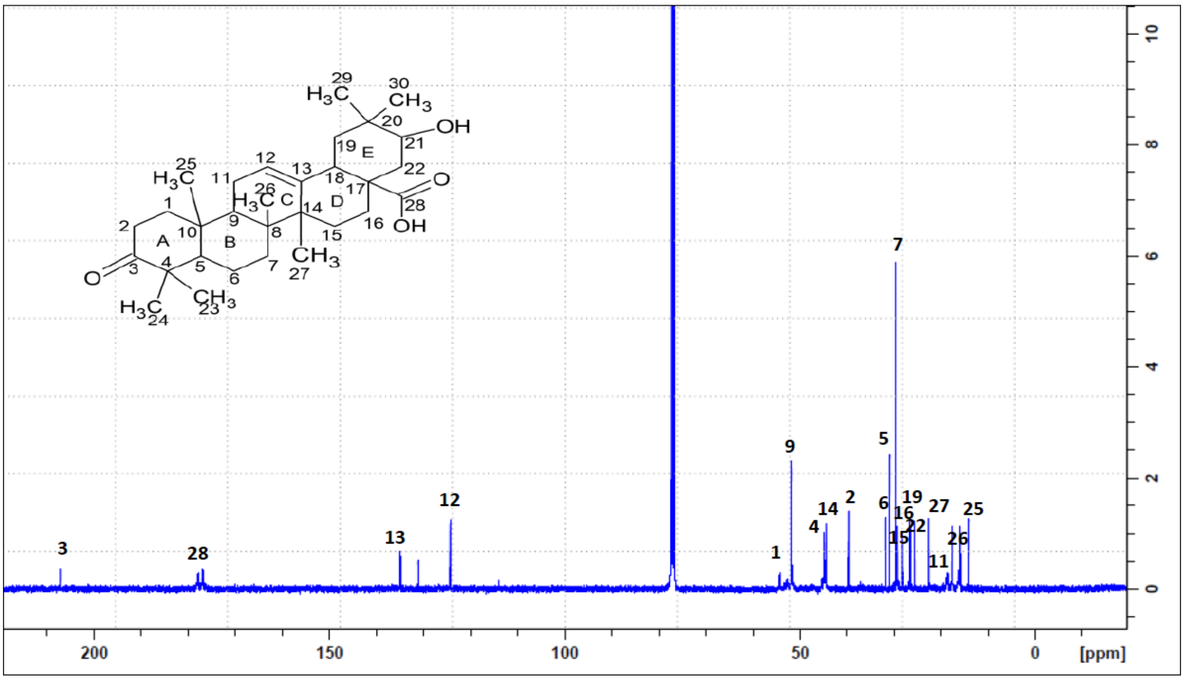

Figure S2. ^{13}C-NMR spectrum of compound **B** showing similarities with reported literature data

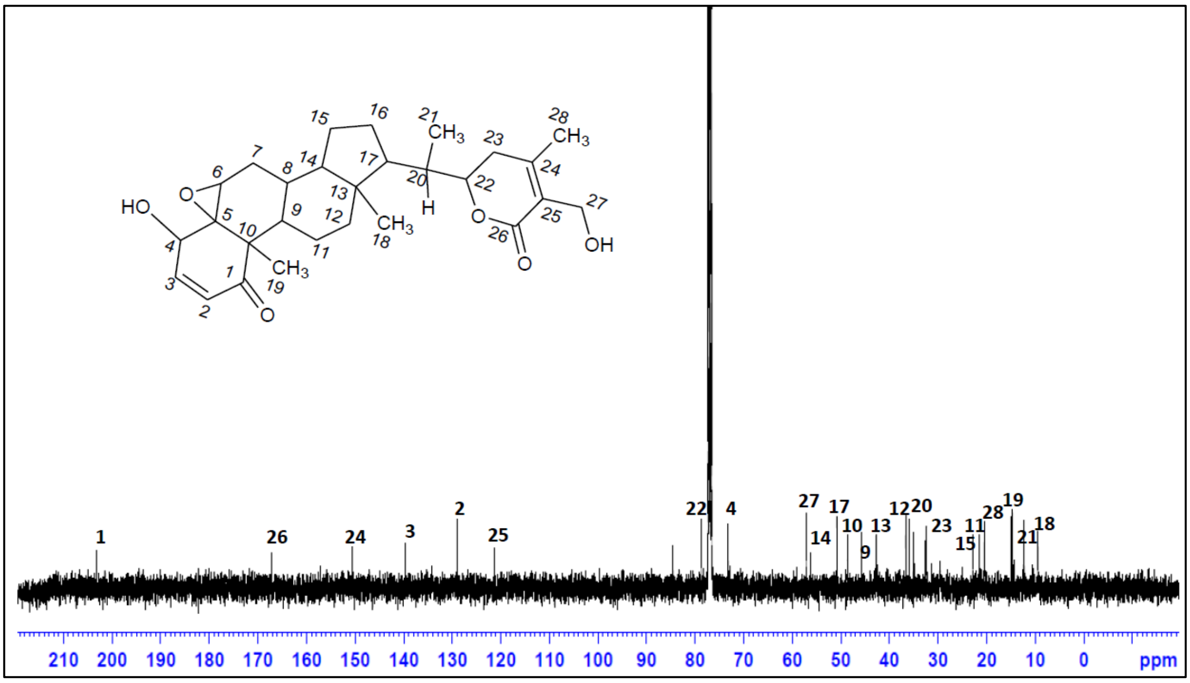

Figure S3. ^{13}C-NMR spectrum of withanolide compound isolated from *W. somnifera* leaf ethyl acetate extract.

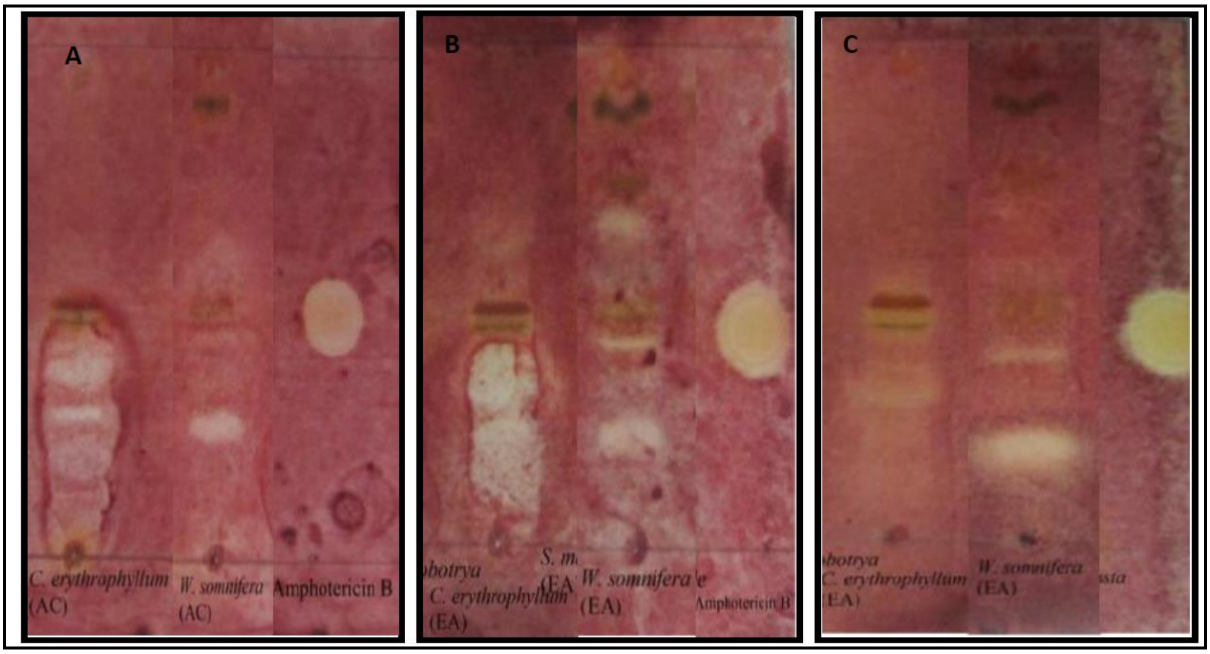

Figure S4. Thin Layer Chromatography (TLC) Bioautography of *Combretum erythrophyllum* and *Withania somnifera* extracts treated with different *Fusarium* pathogens. (**A**): Antifungal activity of *C. erythrophyllum* and *W. somnifera* acetone extracts against *F. verticilloides*. (**B**): Antifungal activity of *C. erythrophyllum* and *W. somnifera* ethyl acetate extracts against *F. proliferatum*. (**C**): Antifungal activity of *C. erythrophyllum* and *W. somnifera* ethyl acetate extracts against *F. solani.* Amphotericin B was included as positive control.

© 2020 by the authors. Submitted for possible open access publication under the terms and conditions of the Creative Commons Attribution (CC BY) license (http://creativecommons.org/licenses/by/4.0/).
